# Selection of X-ray Tube Settings for Relative Bone Density Quantification in the Knee Joint of Cats Using Computed Digital Absorptiometry

**DOI:** 10.3390/s24175774

**Published:** 2024-09-05

**Authors:** Joanna Bonecka, Bernard Turek, Krzysztof Jankowski, Marta Borowska, Tomasz Jasiński, Katarzyna Skierbiszewska, Małgorzata Domino

**Affiliations:** 1 Department of Small Animal Diseases and Clinic, Institute of Veterinary Medicine, Warsaw University of Life Sciences (WULS–SGGW), 02-787 Warsaw, Poland; joanna_bonecka@sggw.edu.pl; 2 Department of Large Animal Diseases and Clinic, Institute of Veterinary Medicine, Warsaw University of Life Sciences (WULS–SGGW), 02-787 Warsaw, Poland; bernard_turek@sggw.edu.pl (B.T.); tomasz_jasinski@sggw.edu.pl (T.J.); katarzyna_skierbiszewska@sggw.edu.pl (K.S.); 3 Institute of Mechanics and Printing, Warsaw University of Technology, 02-524 Warsaw, Poland; krzysztof.jankowski1@pw.edu.pl; 4 Institute of Biomedical Engineering, Faculty of Mechanical Engineering, Białystok University of Technology, 15-351 Bialystok, Poland; m.borowska@pb.edu.pl

**Keywords:** radiography, density standard, densitometry, stifle joint, feline

## Abstract

Bone mineral density (BMD) varies with aging and both systemic and local diseases; however, such evidence is lacking in feline medicine. This may be due to the need for general anesthesia in cats for direct BMD measurements using dual-energy X-ray absorptiometry (DXA) or quantitative computed tomography (QCT). In this study, computed digital absorptiometry (CDA), an indirect relative BMD-measuring method, was optimized to select an X-ray tube setting for the quantitative assessment of the feline knee joint. The knee joints of nine cats were radiographically imaged and processed using the CDA method with an aluminum density standard and five X-ray tube settings (from 50 to 80 kV; between 1.2 and 12 mAs). The reference attenuation of the X-ray beam for ten steps (S1–S10) of the density standard was recorded in Hounsfield units (HU), compared between X-ray tube settings, and used to determine the ranges of relative density applied for radiograph decomposition. The relative density decreased (*p* < 0.0001) with an increase in kV and dispersed with an increase in mAs. Then, the percentage of color pixels (%color pixels), representing ranges of relative density, was compared among S1–S10 and used for the recognition of background artifacts. The %color pixels was the highest for low steps and the lowest for high steps (*p* < 0.0001), regardless of X-ray tube settings. The X-ray tube setting was considered the most beneficial when it effectively covered the lowest possible HU ranges without inducing background artifacts. In conclusion, for further clinical application of the CDA method for quantitative research on knee joint OA in cats, 60 kV and 1.2 mAs settings are recommended.

## 1. Introduction

Bone mineral density (BMD) varies in both human [1,2,3] and feline [4,5,6] populations with aging and various diseases. Feline diseases may affect BMD systemically or locally. Thus, the radiological signs of a decreasing or increasing BMD may be visible throughout the entire skeleton or in specific locations in axial and appendicular regions. Diseases affecting BMD systemically, such as primary and secondary hyperparathyroidism osteopenia [5,7], osteopetrosis [8], osteogenesis imperfecta [9], and mucopolysaccharidosis [10,11], are rare in the feline population. However, diseases affecting BMD locally, such as neoplasia and osteoarthritis (OA), are serious clinical problems. Musculoskeletal neoplasia is uncommonly diagnosed (3.1–4.9 per 100,000 cases), but it is an important differential diagnosis for cats presenting with lameness, pain, or swelling associated with bones and/or soft tissues [12,13,14]. Conversely, OA is a common chronic degenerative form of arthritis with a prevalence in cats ranging from 16% to 91% [15,16,17,18,19,20], with knee joint OA found in as many as 50% of OA-affected joints [18]. Therefore, feline knee joint OA is a further significant target for clinical applied quantitative research on local BMD.

Reports on BMD evaluation in cats have been limited to studies using dual-energy X-ray absorptiometry (DXA or DEXA) [4,10,21,22,23,24], dual-energy quantitative computed tomography (DEQCT) [25], and single-energy quantitative computed tomography (QCT) [5,6,9,22,23], whereas single-energy X-ray computed digital absorptiometry (CDA) has not yet been published for clinical application in cats. Unlike DEQCT, which has so far been used in cats only for the specific examination of mummies [25], DXA was applied in clinically normal cats [4,21,24] and abnormal cats [10,22]. Similarly, QCT was also used in healthy [6,23] and diseased [5,9,22] cats. One may observe that DXA was developed to assess the bone mineral content (BMC) of humans in the context of the diagnosis of osteoporosis [26]; however, both DXA [10,22] and QCT [5,9,22] were successfully used to measure the response of feline bone to systemic diseases affecting BMD, such as mucopolysaccharidosis [10], secondary hyperparathyroidism [5,22], and osteogenesis imperfecta [9]. Therefore, we believe that BMC and BMD quantification can also be successfully used in cats to diagnose diseases affecting BMD locally and to monitor the therapeutic response of musculoskeletal neoplasia and OA.

From the point of view of potential practical use in everyday clinical settings, examining living cats using both DXA and QCT tools required general anesthesia [4,5,6,9,10,22,24] and expensive equipment. The CT scanner [5,6,9,22] is more available in small animal clinics, while the DXA scanner is much less available [4,10,22,24]. Therefore, their application in feline clinical screening and therapy monitoring is limited. In feline clinical practice, conventional radiography does not require general anesthesia and, moreover, serves as the primary method for the initial diagnostic imaging of local bone diseases [12,16,18]. Therefore, we believe that introducing CDA in feline conventional radiography may make relative BMD assessments more useful in clinical practice.

The CDA tool uses a density standard [27,28], also named aluminum markers [29] or radiographic bone aluminum equivalence [30], as a reference for X-ray beam attenuation. This standard allows for the quantification of relative X-ray beam attenuation mediated by the bone and, thus, the bone relative density. By comparing the reference attenuation of the density standard and the relative attenuation of the bone, CDA returns relative BMD, expressed as the number of pixels [27], the percentage of pixels [28], or the brightness/darkness index [29]. Despite the lack of CDA studies in cats, one may note that other veterinary research has measured relative BMD via CDA using 65 kV and 2.5 mAs [27], 60 kV and 1.25 mAs [28], 70 kV and 2.5 mAs [30], and from 55 to 80 kV and from 0.3 to 2.1 mAs [28]. On the other hand, radiographs of cats for the qualitative assessment of knee joint OA were obtained using 50 kV and 2.5 mAs [31] or 2.5–3.2 mAs [32]. Moreover, some studies on the qualitative assessment of feline radiographs do not provide the X-ray tube settings at all [14,15,16,17,18,19,20,33], which significantly increases the risk of bias in such studies. Since X-ray tube settings are crucial for BMD quantification using CDA in the equine third metacarpal bone [29], we hypothesize that in the case of the feline knee joint, the X-ray tube settings affect the relative bone density quantification using the CDA method. This study selected five combinations of tube voltage and current to meet the range from 50 to 80 kV at a low tube current (1.2 mAs) previously studied using the CDA method [27,28,30,31,32]. Additionally, one high tube current (12 mAs) was included, as it is frequently used in feline clinical practice but is not recommended for the CDA method.

Therefore, for the further clinical application of CDA in feline medicine, this study aims to compare the reference attenuation of steps of the density standard among five X-ray tube settings and preliminarily demonstrate the feasibility of relative bone density quantification for the knee joints of cats. As a result of this study, optimal X-ray tube settings will be selected for further quantitative studies on knee joint OA in cats.

## 2. Materials and Methods

### 2.1. Study Design

The prospective observational study was conducted on nine isolated limbs collected from cats after euthanasia. The cats were of European breed (4 neutered females and 5 neutered males), aged 5–12 years. The cats were euthanized for reasons unrelated to the research. The study was performed at the Small Animal Clinic at the Institute of Veterinary Medicine at the Warsaw University of Life Sciences.

### 2.2. Image Acquisition

**Figure 1:**
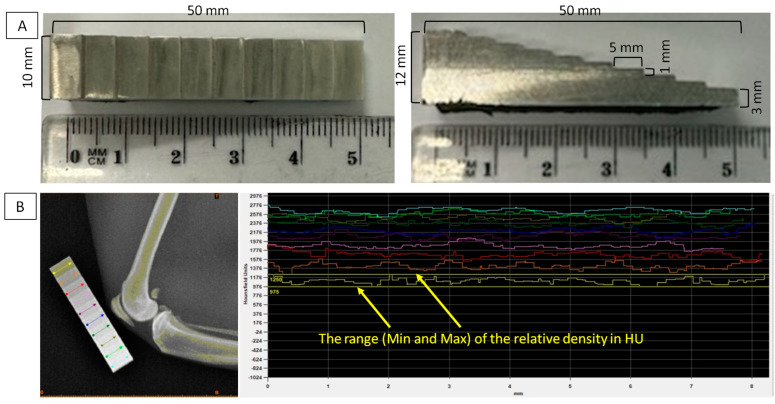
Characteristic of the density standard used. (**A**) Dimensional features: length, height, and width in mm of the entire cuboid and each step (S1–S10), respectively. (**B**) Radiomic features: reference attenuation of the X-ray beam in Hounsfield units (HU) for each step, with the ranges (minimum (Min) and maximum (Max)) of the relative density set for S1 (marked in yellow).

Each isolated limb was imaged using the CDA method in lateral recumbency. The density standard used was aluminum (mass of 9.39 g; density of 2.65 g/cm^3^; aluminum content of 95.20–98.88 by Mass% and 92.71–98.92 by Atom% as detailed in Górski et al. [27]) and had an irregular cuboid shape with 10 steps (S1–S10) decreasing in height (Figure 1A). For X-ray imaging with the CDA method, the density standard was positioned next to the knee joint parallel to the long axis of the patella (Figure 1B). The reference attenuation of the X-ray beam passing through the density standard was measured for each X-ray tube setting using the Materialises Interactive Medical Image Control System (MIMICS) software version 14.0 (Materialise HQ, Leuven, Belgium). The reference attenuation was recorded as a minimum (Min) and maximum (Max) value in Hounsfield units (HU) for each of the 10 steps separately (Figure 1B). These values were measured using a measuring line passing transversely through the center of each step. Measuring lines were positioned from one edge of the density standard to the opposite edge. Based on this, the range of the relative density of each step was determined. These ranges were then used for radiograph decomposition.

**Figure 2:**
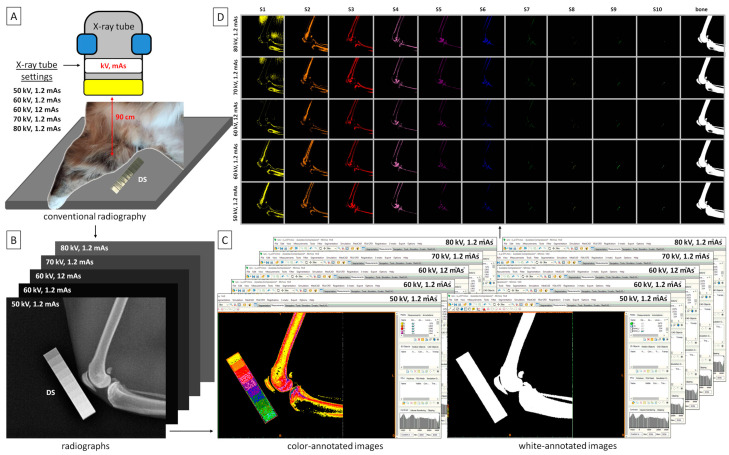
The characteristic of the applied computed digital absorptiometry (CDA) protocol. (**A**) X-ray tube settings used in this study. (**B**) Five radiographs obtained for the respective X-ray tube settings. (**C**) Color-annotated and white-annotated images decomposed from radiographs in relation to the range of the relative density of each step (S1–S10) of the density standard. (**D**) All decomposed images returned for a single knee joint.

Mediolateral radiographs of the knee joint were obtained following the guidelines of Lascelles et al. [18]. The center of the X-ray beam was positioned at the midpoint of the knee joint, and a focus-table distance of 90 cm was set. The X-ray tube settings were changed from 50 to 80 kV and between 1.2 mAs and 12 mAs (Figure 2A), resulting in five radiographs of each knee joint with the following settings: (1) 50 kV and 1.2 mAs; (2) 60 kV and 1.2 mAs; (3) 60 kV and 12 mAs; (4) 70 kV and 1.2 mAs; and (5) 80 kV and 1.2 mAs (Figure 2B). Radiographs were obtained using an X-ray CPI Indico IQ (Communications & Power Industries Canada Inc., Georgetown, ON, Canada) system and acquired digitally in DICOM format using Ubuntu software (Canonical Ltd., Ubuntu Foundation, Isle of Man, London, UK).

### 2.3. Image Processing

Radiographs were decomposed by masking each relative density range (see Section 2.2) with an assigned color (Table 1) following the protocol described by Turek et al. [28]. Decomposition was performed in the MIMICS software version 14.0 (Materialise HQ, Leuven, Belgium) for each X-ray tube setting separately, resulting in color-annotated images. Subsequently, all steps were masked in white (HEX #FFFFFF) to produce white-annotated images (Figure 2C). The total number of pixels in white-annotated images was counted and used as a reference point (bone annotation) for calculating the percentage of each color in the image. Each decomposed image was resized to 899 pixels wide by 847 pixels high and saved as a separate BMP file. This process resulted in each knee joint being represented by eleven decomposed images (ten color-annotated images and one white-annotated image), totaling 55 images for the five different X-ray tube settings (Figure 2D).

### 2.4. Color-Pixel-Counting Protocol

The relative bone density was quantified using the color-pixel-counting protocol described in Turek et al. [28]. Before quantification, the density standard was manually masked using the HEX color #000000 in Paint.NET v.4.3.2 software. Then, the entire non-#000000 pixel area was set as an input area for pixel counting. The CIE76 formula in the color histogram method was used for the calculation of visual similarities. The calculation of color pixels was performed using the extcolors package in Python (https://pypi.org/project/extcolors/, accessed on 19 June 2024). Colors were identified using HEX codes (rgb2hex library; https://colormap.readthedocs.io/en/latest/, accessed on 19 June 2024). The number of pixels of each color was returned from color-annotated images. The number of white pixels was returned from the white-annotated images. The number of white pixels was used for the calculation of the percentage of color-annotated pixels (%color pixels) for each radiograph separately.

### 2.5. Statistical Analysis

The ranges of the relative density were recorded as Min and Max values of the reference attenuation for each step of the density standard and each X-ray tube setting. Each value/step/setting data series consisted of nine realizations. The normality of the data series was tested using the Shapiro–Wilk normality test. Since all data series followed a normal distribution, value/step data series for X-ray tube settings were compared between the X-ray tube settings using the repeated measures ANOVA summary. When *p* < 0.05, the post hoc Holm-Sidak’s multiple comparisons test was used. Due to the normal distribution, data were presented on plots using the mean + standard deviation (SD). The mean ± SD of the Min and Max values of reference attenuation were then summarized in the table.

The %color pixels were recorded for each step of the density standard and each X-ray tube setting, so that each step/setting data series consisted of nine realizations.

The normality of the data series was tested using the Shapiro–Wilk normality test. Since at least one data series was not normally distributed, %color pixels data series were compared between steps of the density standard within X-ray tube setting groups using the Friedman test. When *p* < 0.05, the post hoc Dunn’s multiple comparisons test was used. Due to the non-normal distribution, data were presented on plots using the median and ranges (lower and upper quartiles, as well as minimum and maximum values). The mean ± SD of the %color pixels were then summarized on the plot for subsequent X-ray tube settings, steps of the density standard, and ranges of the relative density.

The statistical analysis was performed using GraphPad Prism v6 (GraphPad Software Inc., San Diego, CA, USA). Statistical significance was set at *p* < 0.05.

## 3. Results

### 3.1. Ranges of the Relative Density

**Figure 3:**
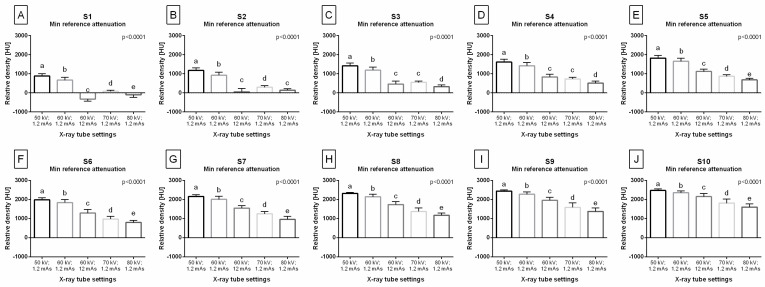
The minimum value (Min) of reference attenuation for each step of the density standard (S1–S10) compared between X-ray tube settings (50 kV; 1.2 mAs; 60 kV; 1.2 mAs; 60 kV; 12 mAs; 70 kV; 1.2 mAs; 80 kV; 1.2 mAs). Plots (**A**–**J**) show the Min reference attenuation for each step: (**A**) Min reference attenuation for S1; (**B**) Min reference attenuation for S2; (**C**) Min reference attenuation for S3; (**D**) Min reference attenuation for S4; (**E**) Min reference attenuation for S5; (**F**) Min reference attenuation for S6; (**G**) Min reference attenuation for S7; (**H**) Min reference attenuation for S8; (**I**) Min reference attenuation for S9; (**J**) Min reference attenuation for S10. The data are presented in Hounsfield units (HU) using the mean + standard deviation (SD). Lowercase letters indicate differences between the X-ray tube settings. The significance level was set as *p* < 0.05.

The Min value of reference attenuation decreased gradually with the X-ray tube settings, from 50 kV; 1.2 mAs to 80 kV; 1.2 mAs, for the majority steps of the density standard (Figure 3A–J). For S1, the Min reference attenuation was the lowest for 60 kV; 12 mAs, higher for 80 kV; 1.2 mAs, then higher for 70 kV; 1.2 mAs, followed by 60 kV; 1.2 mAs, and the highest for 50 kV; 1.2 mAs (Figure 3A). For S2, the Min reference attenuation was the lowest for 60 kV; 12 mAs and 80 kV; 1.2 mAs, higher for 70 kV; 1.2 mAs, followed by 60 kV; 1.2 mAs, and the highest for 50 kV; 1.2 mAs (Figure 3B). For S3 (Figure 3C) and S4 (Figure 3D), the Min reference attenuation was the lowest for 80 kV; 1.2 mAs, higher for 60 kV; 12 mAs and 70 kV; 1.2 mAs, followed by 60 kV; 1.2 mAs, and the highest for 50 kV; 1.2 mAs. For S5 (Figure 3E), S6 (Figure 3F), S7 (Figure 3G), S8 (Figure 3H), S9 (Figure 3I), and S10 (Figure 3J), the Min reference attenuation was the lowest for 80 kV; 1.2 mAs, higher for 70 kV; 1.2 mAs, followed by for 60 kV; 12 mAs, then higher for 60 kV; 1.2 mAs, and the highest for 50 kV; 1.2 mAs.

**Figure 4:**
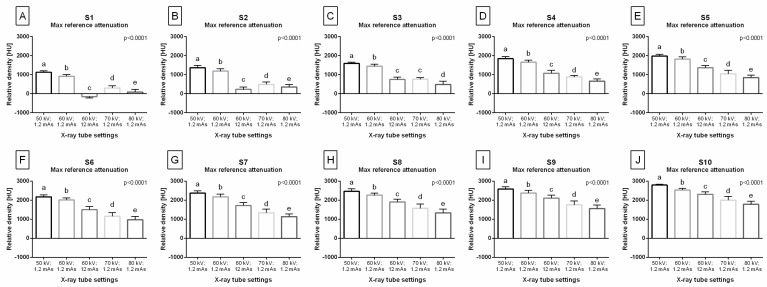
The maximum value (Max) of reference attenuation for each step of the density standard (S1–S10) compared between X-ray tube settings (50 kV; 1.2 mAs; 60 kV; 1.2 mAs; 60 kV; 12 mAs; 70 kV; 1.2 mAs; 80 kV; 1.2 mAs). Plots (**A**–**J**) show the Max reference attenuation for each step: (**A**) Max reference attenuation for S1; (**B**) Max reference attenuation for S2; (**C**) Max reference attenuation for S3; (**D**) Max reference attenuation for S4; (**E**) Max reference attenuation for S5; (**F**) Max reference attenuation for S6; (**G**) Max reference attenuation for S7; (**H**) Max reference attenuation for S8; (**I**) Max reference attenuation for S9; (**J**) Max reference attenuation for S10. The data are presented in Hounsfield units (HU) using the mean + standard deviation (SD). Lowercase letters indicate differences between the X-ray tube settings. The significance level was set as *p* < 0.05.

The Max value of reference attenuation also decreased gradually with the X-ray tube settings, from 50 kV; 1.2 mAs to 80 kV; 1.2 mAs, for all steps of the density standard (Figure 4A–J). For S1 (Figure 4A) and S2 (Figure 4B), the Max reference attenuation was the lowest for 60 kV; 12 mAs, higher for 80 kV; 1.2 mAs, then higher for 70 kV; 1.2 mAs, followed by for 60 kV; 1.2 mAs, and the highest for 50 kV; 1.2 mAs. For S3, the Max reference attenuation was the lowest for 80 kV; 1.2 mAs, higher for 60 kV; 12 mAs and 70 kV, then higher for 60 kV; 1.2 mAs, and the highest for 50 kV; 1.2 mAs (Figure 4C). For S4 (Figure 4D), S5 (Figure 4E), S6 (Figure 4F), S7 (Figure 4G), S8 (Figure 4H), S9 (Figure 4I), and S10 (Figure 4J), the Max reference attenuation was the lowest for 80 kV; 1.2 mAs, higher for 70 kV; 1.2 mAs, then higher for 60 kV; 12 mAs, followed by 60 kV; 1.2 mAs, and the highest for 50 kV; 1.2 mAs.

The Min and Max values of reference attenuation are summarized in Table 2 to present the approximate ranges of the relative densities for which %color pixels were quantified in the next step. The ranges presented in Table 2 are shown as mean ± SD values, whereas the ranges used for %color pixel quantification were calculated individually for each radiograph.

### 3.2. Relative Bone Density Quantification

**Figure 5:**
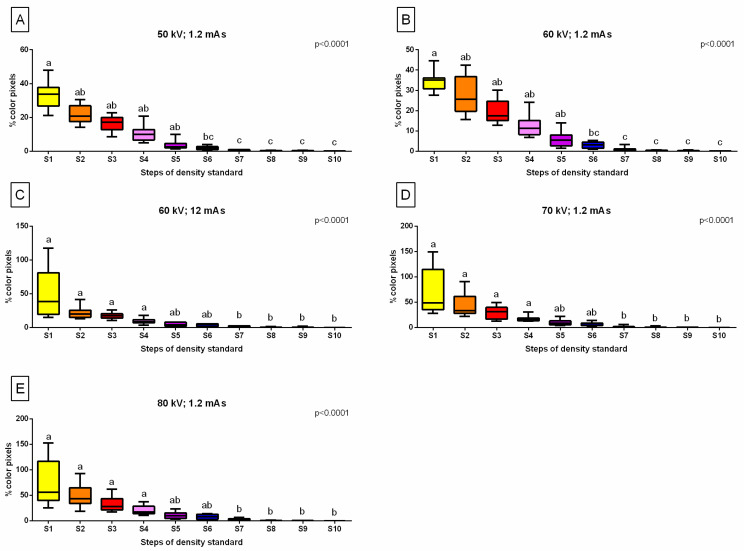
The percentage of color-annotated pixels (%color pixels) for each X-ray tube setting ((**A**) 50 kV; 1.2 mAs; (**B**) 60 kV; 1.2 mAs; (**C**) 60 kV; 12 mAs; (**D**) 70 kV; 1.2 mAs; (**E**) 80 kV; 1.2 mAs) compared between steps of the density standard (S1–S10). Boxes represent the lower quartile, median, and upper quartile, whereas whiskers represent minimum and maximum values. Lowercase letters indicate the differences among S1–S10. The significance level was set as *p* < 0.05.

The %color pixels decreased gradually with the steps of the density standard, from S1 to S10, for all settings of the X-ray tube (Figure 5A–E). For 50 kV; 1.2 mAs (Figure 5A) and 60 kV; 1.2 mAs (Figure 5B), and the %color pixels was higher for S1–S5 than for S7–S10, with no differences between S2–S6 and S6–S10. For 60 kV; 12 mAs (Figure 5C), 70 kV; 1.2 mAs (Figure 5D), and 80 kV; 1.2 mAs (Figure 5E), the %color pixels were higher for S1–S4 than for S7–S10, with no differences between S1–S6 and S5–S10.

**Figure 6:**
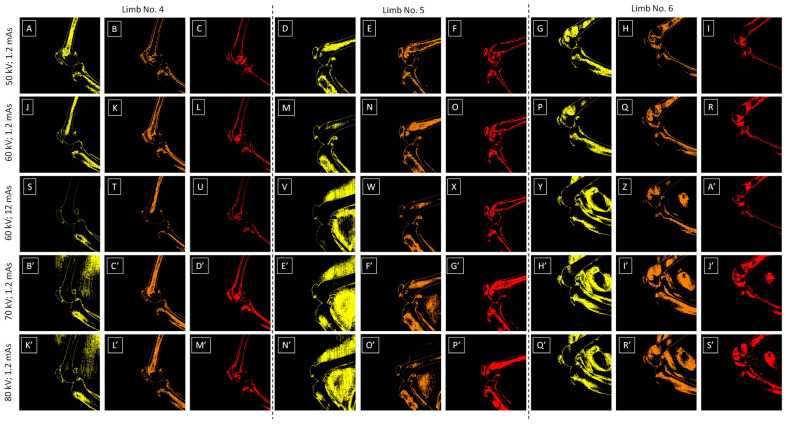
Sample color-annotated images with varying degrees of background artifacts. These images were obtained using the following X-ray tube settings: (**A**–**I**) 50 kV; 1.2 mAs, (**J**–**R**) 60 kV; 1.2 mAs, (**S**–**A’**) 60 kV; 12 mAs, (**B’**–**J’**) 70 kV; 1.2 mAs, and (**K’**–**S’**) 80 kV; 1.2 mAs. These images were decomposed for the following steps (S1–S3) of the density standard: (**A**,**D**,**G**,**J**,**M**,**P**,**S**,**V**,**Y**,**B’**,**E’**,**H’**,**K’**,**N**,**Q’**) S1, (**B**,**E**,**H**,**K**,**N**,**Q**,**T**,**W**,**Z**,**C’**,**F’**,**I’**,**L’**,**O’**,**R’**) S2, and (**C**,**F**,**I**,**L**,**O**,**R**,**U**,**X**,**A’**,**D’**,**G’**,**J’**,**M’**,**P’**,**S’**) S3. Dashed lines separate image groups of the same limbs: (**A**–**C**,**J**–**L**,**S**–**U**,**B’**–**D’**,**K’**–**M’**) limb No. 4, (**D**–**F**,**M**–**O**,**V**–**X**,**E’**–**G’**,**N’**–**P’**) limbs No. 5, and (**G**–**I**,**P**–**R**,**Y**–**A’**,**H’**–**J’**,**Q’**–**S’**) limbs No. 6, which are characterized by the presence of mild, moderate, and severe background artifacts, respectively.

Considering the ordinate representing %color pixels, one may observe that for 60 kV; 12 mAs (Figure 5C), 70 kV; 1.2 mAs (Figure 5D), and 80 kV; 1.2 mAs (Figure 5E), the scale exceeded 100% due to the presence of background artifacts. Examples of background artifacts and the corresponding artifact-free images are presented in the Figure 6A–S’. One may observed that all color-annotated images for 50 kV; 1.2 mAs (Figure 6A–I) and 60 kV; 1.2 mAs (Figure 6J–R) were artifact-free, whereas in some images for 60 kV; 12 mAs (Figure 6S–A’), 70 kV; 1.2 mAs (Figure 6B’–J’), and 80 kV; 1.2 mAs (Figure 6K’–S’), mild, moderate, and severe background artifacts were observed. Mild background artifacts were only observed for step S1 and 70 kV; 1.2 mAs/80 kV; 1.2 mAs settings (Figure 6B’,K’). Moderate background artifacts were observed for step S1 and 60 kV; 12 mAs/70 kV; 1.2 mAs/80 kV; 1.2 mAs settings, as well as for step S2 and 70 kV; 1.2 mAs/80 kV; 1.2 mAs settings (Figure 6V,E’,F’,N’,O’). Severe background artifacts were observed for step S1 and 60 kV; 12 mAs/70 kV; 1.2 mAs/80 kV; 1.2 mAs settings, step S2 and 60 kV; 12 mAs/70 kV; 1.2 mAs/80 kV; 1.2 mAs settings, as well as step S3 and 70 kV; 1.2 mAs/80 kV; 1.2 mAs settings (Figure 6Y,Z,H’,I’,J’,Q’,R’,S’). Background artifacts were only observed for steps S1–S3 and were not observed for steps S4–S10.

**Figure 7:**
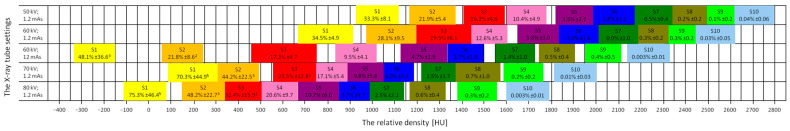
The plot shows the relation of %color pixels (mean ± SD) among X-ray tube settings (50 kV; 1.2 mAs; 60 kV; 1.2 mAs; 60 kV; 12 mAs; 70 kV; 1.2 mAs; 80 kV; 1.2 mAs), steps of the density standard (S1–S10), and ranges of the relative density in Hounsfield units (HU). Numbers in superscripts indicate measurements in which background artifacts occurred, including the following: ^2^ background artifacts in 2/9 measurements; ^3^ background artifacts in 3/9 measurements; and ^5^ background artifacts in 5/9 measurements.

Since background artifacts were observed in some, but not all, images, five image groups (Limbs No. 3–7) showed various severities of background artifacts, whereas four image groups (Limbs No. 1–2 and 8–9) were considered artifact-free. For the image groups of two limbs (Limbs No. 3 and 4), mild background artifacts were noted. For the image group of one limb (Limbs No. 5), moderate background artifacts were noted. For image groups of the other two limbs (Limbs No. 6 and 7), severe background artifacts were noted. Summarizing, for step S1 and 60 kV; 12 mAs settings, three background artifacts were noted; for step S2 and 60 kV; 12 mAs settings two background artifacts were noted; for step S1 and 70 kV; 1.2 mAs/80 kV; 1.2 mAs settings five background artifacts were noted for each, for step S2 and 70 kV; 1.2 mAs/80 kV; 1.2 mAs settings, three background artifacts were noted for each, and for step S3 and 70 kV; 1.2 mAs/80 kV; 1.2 mAs settings, two background artifacts were noted for each, as indicated by superscripts in Figure 7.

In the plot presented in Figure 7, the relation between %color pixels between X-ray tube settings and ranges of the relative density are summarized for all steps of the density standard. Each colored box on the plot represents the color and HEX code assigned to the consecutive steps of the density standard. The range of each colored box covers the respective range of the relative density and is supported by the %color pixels and the number of occurrences of background artifacts. For 50 kV; 1.2 mAs settings, the ranges of relative density start at approximately 880 HU and cover almost the entire range of relative densities up to approximately 2800 HU, with no background artifacts observed. For 60 kV; 1.2 mAs settings, the ranges of relative density start at approximately 680 HU and end at approximately 2500 HU, also covering almost the entire range of relative densities with no background artifacts. For 60 kV; 12 mAs settings, the ranges of relative density start at approximately −330 HU and end at approximately 2300 HU. However, both scale dispersion and lack of full relative density range coverage and background artifacts are visible. For 70 kV; 1.2 mAs settings, the ranges of relative density start at approximately 90 HU and cover almost the entire range of relative densities up to approximately 2000 HU, with background artifacts. For 80 kV; 1.2 mAs settings, the ranges of relative density start at approximately −100 HU and end at approximately 1800 HU, also covering almost the entire respective range. However, also background artifacts are observed. In this summary, one may observe the shift in color annotations from negative HU values for 60 kV; 12 mAs and 80 kV; 1.2 mAs settings to the highest positive HU values for the 50 kV; 1.2 mAs setting. The coverage of relative density ranges is adjacent for 50 kV–80 kV; 1.2 mAs and increases on the HU scale from 50 kV; 1.2 mAs to 80 kV; 1.2 mAs. For 60 kV; 12 mAs, one can notice a scattering of coverage of relative densities with the successive steps (Figure 7).

## 4. Discussion

The obtained results allow us to accept the hypothesis of this research, showing that the X-ray tube settings affect the relative bone density quantification of the feline knee joint when using the CDA method. This finding is consistent with Bower et al.’s [29] research on the equine third metacarpal bone, which indicate that calibrating of radiographic opacity with various X-ray tube settings enables the quantification of BMD. The authors developed a method based on standardized digital radiographs that allows for an accurate measurement of BMD [29]. A variant of this developed method was tested in this study on feline specimens.

Bowers et al. [29] assumed that the quantified region of interest (ROI) should include minimal soft tissue and possess consistent thickness and attenuation properties. While feline knees may vary in thickness in the mediolateral view, the aluminum density standard used has a known thickness, density, and molecular composition [28], ensuring consistent attenuation properties. Bowers et al. [29] used an aluminum wedge standard of a known density and increasing thickness considered equivalent of BMD and demonstrated a linear correlation between the relative density of the aluminum standard and bone, independent of the exposure setting. In this study, a decrease in %color pixels was observed with an increasing thickness of the density standard (through subsequent steps), also regardless of the exposure setting.

Since BMD primarily depends on the thickness and density of the imaged structure [29], comparing ROIs with higher or lower BMD to other ROIs of similar bone and soft tissue thickness allows for conclusions regarding local densities. This relationship and comparison may have practical applications in assessing the occurrence, progression, and treatment response of feline OA [18,32,33], especially since tissue thickness, including the individual amount of soft tissue, remains constant when monitoring the same cat. Interestingly, DXA can also evaluate BMC as a percentage of body weight [4,21] or in grams across the entire body [24], making it a tool for evaluating the overall body condition of cats and not just the skeletal status. In this preliminary study, the entire knee joint was designated for color pixel counting, and manual image segmentation was not applied. While this approach suffices for selecting optimal X-ray tube settings, it limits the feasibility of the protocol to quantify specific radiological signs of knee joint OA [32,33] as listed above. Therefore, the preliminary methodology requires expansion through knee joint structure segmentation and testing on a larger group of cats with and without confirmed OA. Unlike studies requiring the use of general anesthesia [4,5,6,9,10,22,24], which Cheon et al. [6] noted can hinder recruitment, clinical trials using CDAs will not require general anesthesia. Therefore, CDAs can be integrated into standard feline clinical practice, providing a robust radiographic database from numerous patients.

The feline knee joint composes dense cortical bone, less dense trabecular bone, and little soft tissue, mainly consisting of skin, ligaments, and fat [34,35], with radiographic density ranges from 700 to 2500 HU for bone, ≥350 for mineralization, and from 10 to 40 HU for soft tissue [31,36,37]. One may observe that on the reference HU scale at a standard temperature and pressure, the radiodensity increases with the density of the imaged material and is defined as −1000 HU for air, 0 HU for distilled water, 20–100 HU for soft tissue, up to 1000 HU for bone, and up to 3000 HU for dense bone or tooth [36]. This is because attenuation of the X-ray beam is heavily dependent on the atomic number of the tissues and increases with the density of the imaged material [38]. The summary plot illustrates that it is not possible to cover the full range of the HU scale with only ten steps of the density standard. Given the specific range available, it is desirable to have a range of relative densities that covers the range of tissues undergoing quantification.

This range, as depicted in Figure 7, is adequately covered in the lower part by 70 kV; 1.2 mAs and 80 kV; 1.2 mAs settings and in the upper part by 50 kV; 1.2 mAs and 60 kV; 1.2 mAs settings, with the upper part appearing to be more advantageous due to better alignment with the bone HU range [31,36,37]. The tube voltage governs the energy of the X-ray beam and their ability to penetrate through tissue. The higher the kV, the greater the energy of the X-ray beam and the greater their ability to penetrate imaged material, with both density standard and tissues undergoing quantification [38]. One may observe that increasing the kV in the X-ray tube settings reduces the reference attenuation of the aluminum density standard, thereby shifting the relative density ranges used for quantifying relative bone density towards the soft tissue range. Soft tissues, such skin, ligaments, and fat, absorb limited numbers of X-ray beams, even of a low kV, while bone absorbs more X-ray beams of a low kV than high kV [38]. Therefore, there is a significant difference in the number of X-ray beams passing through the soft tissues than bone when a low kV is used. A low kV produces a high-contrast image with a low-exposure latitude, while high produces a low-contrast image with a wider exposure latitude [38]. Hence, images obtained using 50 kV; 1.2 mAs and 60 kV; 1.2 mAs settings may be characterized by the relatively high contrast and therefore better determination of ranges of relative density and a lower occurrence of background artifacts. On the other hand, a higher tube current results in a greater number of photons being produced in the X-ray beam, while the quantity of photons reaching the detector affects image opacity [38]. Therefore, increasing the mAs in the X-ray tube settings causes the relative density ranges to disperse, increasing the distance between the reference attenuation ranges of individual steps of the aluminum density standard and shifting the lowest steps further toward the soft tissue range. These shifts, while potentially beneficial for assessing soft tissue, are also associated with the occurrence of background artifacts in some of the quantified radiographs.

The occurrence of background artifacts results from digital image processing, which, as noted by Bowers et al. [29], can potentially affect the measurement of BMD and warrants further investigation for clinical applicability. In the image processing method applied in this study, the image decomposition algorithm identifies a portion of the background as representing the same gray scale as the lower steps (S1–S3) of the density standard, particularly evident for X-ray tube settings that shift the relative density ranges towards lower HU values (60 kV; 12 mAs; 70 kV; 1.2 mAs; 80 kV; 1.2 mAs). Since air transmits rather than attenuates most of the X-ray beam [39], areas near the limb, especially where the hair of long-haired cats is present, are decomposed similarly to the soft tissues. Consequently, background artifacts do not arise for higher steps of the density standard (S4–S10) and for X-ray tube settings that shift the relative density ranges towards higher HU values (50 kV; 1.2 mAs; 60 kV; 1.2 mAs).

### Limitations

The limitation related to background artifacts can be addressed using the flat-field correction [40,41] or by the segmentation of the radiographs and focusing only on ROIs corresponding to specific bone areas for the quantification of relative BMD, such as segmenting the distal femur, proximal tibia, and patella as separate ROIs [42]. This segmentation, in future studies of feline knee joint OA, will enable the quantification of radiological signs of OA and assessments of OA severity. Mild OA is characterized by a narrow and irregular joint space with osteophytes, while moderate OA shows a narrow and irregular joint space with multiple osteophytes, enthesiophytes, and moderate intra-articular mineralization. Severe OA presents with a completely narrowed joint space, large osteophytes and enthesiophytes, and severe intra-articular mineralization [32]. Further development of the described protocol towards radiograph segmentation is justified, as reference methods for BMD assessments also return measured values for selected ROIs. QCT provides BMD as the mean HU [6,9,22] or mineral equivalents (mg/mL) [5] in specific ROIs. Recent feline QCT studies have set ROIs in various areas, such as the lumbar vertebrae (L1–L5), femur, tibia, pelvis [5,22], thoracic (Th12–Th13) and lumbar (L1–L7) vertebrae and ilium [6], and L1–L7 vertebrae [9]. Moreover, one DXA study in cats returned BMD and BMC values for ROIs set at the level of the L5–L6 [10], and the second DXA study returned BMD for ROIs set in the femur, tibia, and pelvis [22]. However, no studies to date have returned measures of bone density, either relative or absolute, for ROIs set in the distal femur, proximal tibia, and patella, neither in healthy nor diseased cats.

In further applications, the limitations caused by the transmission of the X-ray beam through tissues must be addressed. Since pixel intensity depends on the material composition, density, and transmission length [38], the thickness of the imaged area should be considered. To mitigate this issue, it would be advisable to measure the width of the distal femur, proximal tibia, and patella and use these thickness measurements to calculate the corrected BMD. These measurements justify the need to segment individual ROIs [42] and simultaneously image the same structures using CT, as CT allows for accurate bone size measurements [43]. Additionally, simultaneous imaging with both CDA and CT or CDA and DXA would help to verify the accuracy of the relative bone density assessment. Such a study would demonstrate whether decomposing the X-ray image based on intensity behind the aluminum density standard is justified in felines, as has been shown in horses [29,30]. Bowere et al. [29] calibrated the CDA method and demonstrated a linear relation between the brightness/darkness index (BDI) (pixel intensity) of bone and the aluminum density standard, considering that the CDA method can be used for the quantitative, noninvasive bone mineral analysis of the equine third metacarpal bone. At this stage of feline research, the absence of reference measurements is a significant limitation, necessitating further research to address this gap.

Moreover, it is important to note that pixel intensity is influenced by the cumulative attenuation along the corresponding X-ray beam [38], which propagates through cortical bone, trabecular bone, and cortical bone again. Therefore, future work should take into account not only ROI segmentation but also the detailed anatomy of the cat knee, which has been covered in only a few publications [34,35]. We believe that in further studies, comparing measurements between the same anatomical structures in different cats or within the same cat over subsequent imaging sessions, while considering the individual thickness of the structures being compared, will be a better approach than comparing different structures, such as the distal femur and proximal tibia. Despite this limitation, the CDA method still holds clinical potential, for instance, in monitoring the progression of knee joint OA [18,19,20], by matching pixel intensity to the equivalent thickness of aluminum [29,44]. However, further studies are needed in which the shape and size of the investigated bones are comparable and the ROIs of healthy bone and bone with higher mineralization due to OA are compared. Simultaneously comparing CDA radiography results with those obtained using CT or DAX as reference methods will be essential for determining the CDA method’s clinical utility.

However, this study aims to demonstrate the effect of lamp settings on imaging quality. At this stage, we can confidently state that the bones of feline knee joints absorb X-ray beams in a similar range as the density standard when low tube voltage and current settings are used. These X-ray tube settings are considered the most beneficial for relative bone density quantification using the CDA method, as they effectively cover the lowest possible HU ranges without introducing background artifacts. These low settings are recommended for further comparative studies, which are needed as a next step to gradually introduce the CDA method into feline clinical practice and significantly expand the current diagnostic possibilities of feline bone diseases.

## 5. Conclusions

The X-ray tube settings affect the relative bone density quantification of the feline knee joint using the CDA method. Increasing the kV reduces the reference attenuation of the aluminum density standard, thereby shifting the relative density ranges towards the lower HU values. Increasing mAs causes the relative density ranges to disperse, shifting the ranges further toward the lower HU values. Both of these shifts are incidentally associated with the occurrence of background artifacts. Therefore, among the studied X-ray tube settings, the most beneficial and recommended for further clinical applied quantitative research on knee joint OA in cats using the CDA method is 60 kV and 1.2 mAs.

## Figures and Tables

**Table 1:** The colors and HEX codes assigned to the ten steps of the density standard (S1–S10).

Decomposition	S1	S2	S3	S4	S5	S6	S7	S8	S9	S10
Color	Yellow	Orange	Red	Light purple	Dark purple	Dark blue	Dark green	Navy green	Light green	Light blue
HEX code	#FFFF00	#E08000	#FF0000	#E080C0	#800080	#0000FF	#008000	#808000	#00FF00	#A6CAF0

**Table 2:** The ranges (mean ± SD of the minimum (Min) and maximum (Max) values) of reference attenuation reflecting the relative density of ten steps of the density standard (S1–S10) were summarized for each X-ray tube setting used (50 kV; 1.2 mAs; 60 kV; 1.2 mAs; 60 kV; 12 mAs; 70 kV; 1.2 mAs; 80 kV; 1.2 mAs) in Hounsfield units (HU)).

X-ray Tube Settings	Range	S1	S2	S3	S4	S5	S6	S7	S8	S9	S10
50 kV; 1.2 mAs	Min	880.6 ± 129.0	1173.9 ± 143.5	1417.8 ± 147.2	1614.4 ± 155.8	1826.1 ± 142.2	1985.0 ± 109.0	2157.2 ± 104.0	2318.9 ± 60.1	2447.2 ± 56.8	2478.3 ± 90.8
	Max	1130.6 ± 69.6	1367.8 ± 122.3	1590.8 ± 70.8	1841.7 ± 112.4	1982.8 ± 93.9	2182.8 ± 99.7	2379.4 ± 111.4	2470.6 ± 126.5	2588.9 ± 116.2	2798.9 ± 47.4
60 kV; 1.2 mAs	Min	672.8 ± 142.5	928.9 ± 155.9	1194.4 ± 154.6	1423.9 ± 163.3	1658.9 ± 157.7	1848.3 ± 158.0	2012.8 ± 157.7	2149.4 ± 139.6	2280.6 ± 129.0	2363.3 ± 96.8
	Max	919.4 ± 88.3	1191.7 ± 122.1	1446.7 ± 103.9	1659.4 ± 112.8	1821.1 ± 115.0	2018.9 ± 104.8	2181.1 ± 142.7	2268.3 ± 107.1	2381.1 ± 143.7	2539.4 ± 94.0
60 kV; 12 mAs	Min	−330.0 ± 104.1	63.3 ± 170.8	465.0 ± 148.5	837.8 ± 137.7	1125.6 ± 116.9	1291.9 ± 195.1	1554.4 ± 133.8	1741.1 ± 149.6	1965.6 ± 159.1	2156.7 ± 166.1
	Max	−148.6 ± 68.3	231.1 ± 120.4	747.8 ± 119.7	1078.9 ± 150.4	1365.0 ± 115.1	1507.2 ± 156.3	1727.2 ± 158.9	1910.0 ± 142.2	2115.0 ± 156.3	2306.7 ± 143.2
70 kV; 1.2 mAs	Min	86.1 ± 52.2	294.7 ± 78.7	546.7 ± 78.8	732.9 ± 81.9	871.1 ± 80.5	976.7 ± 148.1	1255.6 ± 122.5	1379.4 ± 187.2	1590.6 ± 240.1	1815.0 ± 212.1
	Max	302.8 ± 120.2	490.0 ± 128.7	762.8 ± 82.4	885.0 ± 80.2	1045.6 ± 183.3	1161.1 ± 189.9	1342.8 ± 196.3	1570.0 ± 235.9	1760.6 ± 207.5	2007.2 ± 196.1
80 kV; 1.2 mAs	Min	−103.3 ± 133.6	140.6 ± 83.1	328.3 ± 95.9	513.3 ± 102.6	682.2 ± 83.8	797.2 ± 115.3	960.0 ± 156.9	1177.8 ± 119.3	1380.0 ± 177.3	1608.9 ± 165.9
	Max	92.2 ± 135.9	359.4 ± 121.1	487.8 ± 178.1	663.9 ± 117.6	850.6 ± 130.9	975.6 ± 179.3	1142.2 ± 135.2	1339.4 ± 201.2	1563.9 ± 185.7	1786.7 ± 162.3

## Data Availability

The data presented in this study are available upon request from the corresponding author.
